# Molecular Adaptations of Thermophilic Proteins—From Mechanistic Understanding to Machine Learning Approaches

**DOI:** 10.1002/open.70234

**Published:** 2026-07-13

**Authors:** Sermarajan Arunachalam, Balamurali MM, Ramachandran Gnanasekaran

**Affiliations:** ^1^ Department of Chemistry School of Advanced Sciences, Vellore Institute of Technology Chennai India; ^2^ Centre for Healthcare Advancement, Innovation and Research Vellore Institute of Technology Chennai India

**Keywords:** artificial intelligence, deep learning, machine learning, mesophiles, mutational studies, protein engineering, protein folding, thermophiles

## Abstract

Thermophilic proteins are known for their stability and activity at elevated temperatures and hence serve as useful models for engineering enzymes with enhanced stability. The stability of these proteins is governed by both thermodynamic and kinetic factors that affect the folding–unfolding equilibrium and the rate of irreversible denaturation. Structural studies have shown that enhanced thermostability often results from multiple cooperative interactions, including improved hydrophobic packing, strengthened electrostatic interactions, and optimized hydrogen‐bonding networks. Comparative studies between thermophilic and mesophilic proteins have helped to identify general mechanisms that support thermal adaptations. Mutational and protein‐engineering approaches have further demonstrated the influence of specific amino acid residues or motifs on the stability through altered or modified local structural interactions. In recent years, computational approaches and machine learning methods have emerged as inevitable tools to predict protein stability. Although these approaches have improved the predictions, there still exist several limitations like limited training datasets, bias toward certain protein families, difficulties in model interpretation, etc. Therefore, combining experimental thermodynamic data, structural studies, and modern AI‐based computational tools, it is possible to develop more reliable strategies to design thermostable proteins, in particular, for various industrial and biotechnological applications.

## Graphical abstract

Several factors including hydrophobic packing, electrostatic interactions and hydrogen bonding influence thermostability of proteins. Mechanisms of thermal adaptation were investigated via structural and mutational studies. Stability predictions were enhanced by combining thermodynamic data with ML‐based computer models for protein engineering.

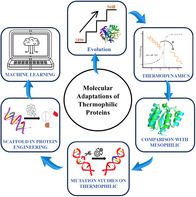

## 1 Introduction

Organisms that thrive in extreme environmental conditions like high temperature, pressure, acidity and alkalinity are referred to as extremophiles [1]. Among these extremophiles, thermophiles are unique, as their optimal biological growth occurs in the temperature range of 50°C–100°C. The inherent molecular mechanisms in these thermophilic organisms enable them to survive and operate effectively at elevated temperatures [2]. The proteins found in such organisms are known as thermophilic proteins. They assist for the enhanced stability and effective functioning even at elevated temperature conditions [3]. The observed stability of these proteins makes their utilization for various other applications including catalytic activities at elevated temperatures [4]. Thermophilic proteins have occupied a strategic position in both biotechnology and chemical processing. Thus, gaining insights into the details of molecular structure and the associated thermostability is significant to address specific stability requirements for manufacturing and pharmaceutical applications [5]. Over the last 10 years, extensive research was conducted to gain better comprehension of the inverse relationship between functionality and stability of proteins. Investigations have revealed that the amino acids that are crucial for enhancing functionality are often stabilizing or destabilizing via various inherited characteristics or the way in which they are positioned within the three‐dimensional protein structure [6]. Despite the fact that thermophilic organisms are usually regarded as nonpathogenic to humans, they might also possess significant economic values. Enzymes derived from thermophiles have exceptional heat stability, making them highly suitable for a wide range of industrial applications [7, 8, 9, 10].

This review primarily focuses on the development of thermophilic proteins within various environments and examines the factors contributing to their thermal stability. The differences between mesophilic and thermophilic proteins, particularly with regard to their amino acid composition and coupling patterns that enhance thermal resilience in thermophiles, are discussed. Also, the recent investigations employing various machine learning (ML) and in silico approaches to identify and assess these thermophilic proteins are also reviewed.

## 2 Evolution of Thermophilic Proteins

**FIGURE 1:**
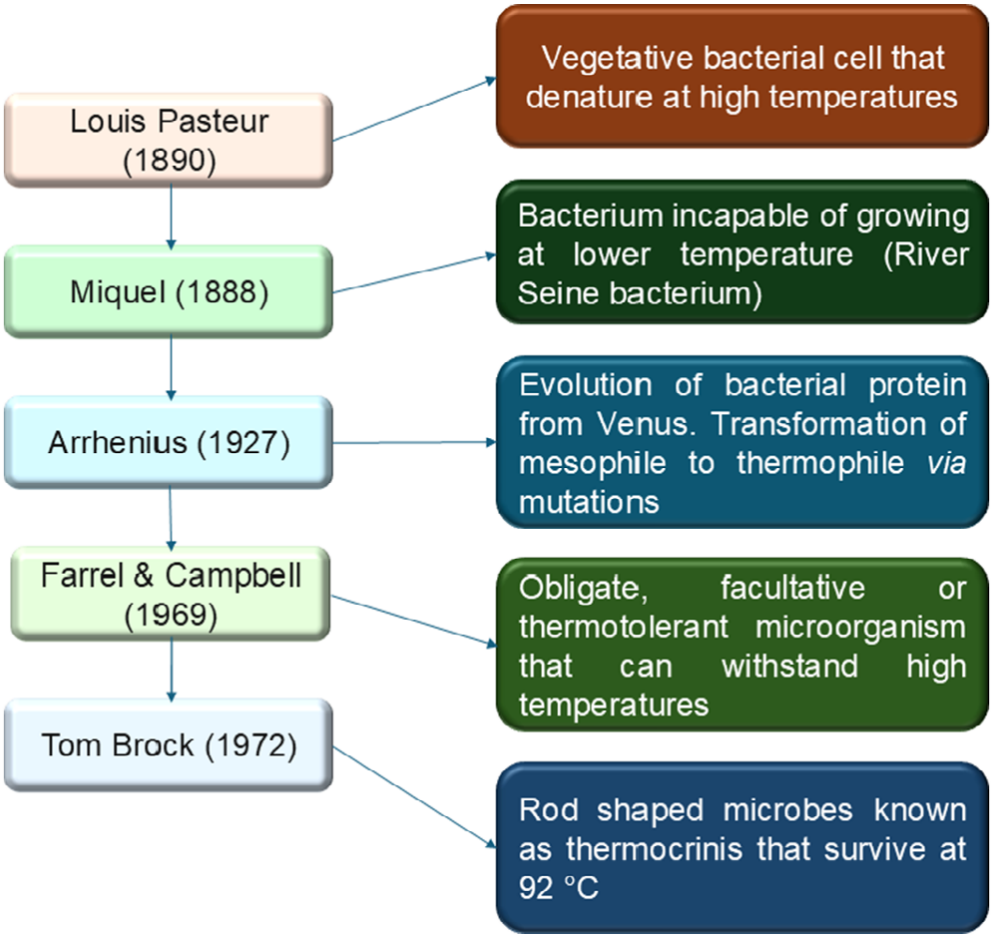
History of developments achieved for thermophilic proteins over the years.

Thermophilic bacteria are diverse group of microorganisms that can thrive under various environments ranging from thermal pools to arctic glaciers. Farrell and Campbell have classified these bacteria as obligate, facultative, or thermally tolerant organisms [11, 12] based on their ability to withstand elevated temperatures. The maximum temperature at which they can survive was theoretically evaluated by Brock [12, 13] and is known to be influenced by the presence of water. The origin of thermophiles is diverse and imaginative since ancient times [12]. Arrhenius suggested a rather exotic origin, proposing that thermophilic microbes originated on Venus and later transported to Earth within few days through radiation and pressure from the sun [14]. There are arguments that favor the mesophilic origin of thermophiles through adaptation or mutation [15] based on the fact of widespread occurrence of thermophilic species in nonthermophilic environments and the observation that certain mesophilic species have adapted to thrive at elevated temperatures. Existing evidences support the thermophilic origin for mesophiles, with the assertion that evolution of thermophiles occurred in an environment that is significantly warmer [16]. A brief summary of the perspectives and classification of thermophilic proteins is shown in Figure 1.

The well‐established concept, originating with Louis Pasteur, asserts the death of vegetative bacterial cells in the temperature range between 80°C and 100°C, which is fundamental to pasteurization. Some thermophiles have revealed an optimal and rapid growth at around 75°C [17] and also follow similar inactivation patterns as mesophiles, with growth ceasing at 100°C. Reports have shown that *Sulfolobus acidocaldarius* can thrive up to 85°C aerobically [17]. Similarly, another rod‐shaped microbe *Thermocrinis* were reported to thrive in boiling hot springs at 92°C at neutral pH [18]. Initially misclassified as *Pseudomonads*, this *Sulfolobus* was thought to have a higher energy yield due to its aerobic lifestyle, which is crucial to withstand thermal destruction [19]. This idea was further supported by the observed decline in growth temperatures in anaerobic thermophilic methanogens.

## 3 Factors Influencing the Thermodynamics of Thermophilic Proteins

The stability of thermophilic proteins is dictated by the free energy change (Δ*G*_FU_) between the folded (F) and unfolded (U) states. While mesophilic and thermophilic proteins often exhibit similar Δ*G*_FU_ at their respective physiological temperatures, their thermodynamic profiles across a temperature range reveal fundamental differences in their adaptation.

Thermophilic proteins generally attain higher *T*_m_ through two primary strategies—(i) reduced Δ*C*_p_ and (ii) enhanced Δ*G* values as temperature increases. The former minimizes the temperature dependence of the unfolding enthalpy and entropy, thereby to exhibiting a wide stability window and hence a shift in *T*_m_ to higher values.

Entropy is another component of thermal adaptation which is often ignored. In thermophiles, entropy of unfolding is influenced by several factors. Enhanced structural rigidity along with a greater number of ion pairs or disulfide bonds in their native states limits conformational diversity of proteins, further diminishing the entropic gain upon unfolding to ultimately stabilize the folded states. Similarly, the hydrophobic interaction in solvent environments, enhances the entropy and lower the overall free energy for protein folding. The temperature at which maximum stability is exhibited, corresponds to zero entropy change, indicating that the enthalpic contributions towards protein. A summary of thermodynamic differences observed between thermophiles and mesophiles is compiled in Table 1.

**TABLE 1:** Summary of influencing factors compared between thermophilic and mesophilic proteins.

Features	Thermophilic proteins	Mesophilic proteins
Ion pair	• More number of ion pairs • Strong ion pair interaction • Ion pairs separated by >4 Å	• Less number of ion pairs • Weak ion pair interaction • Ion pairs separated by 6–8 Å
Hydrophobicity	High hydrophobicity Enhanced residue volume	Low hydrophobicity Reduced residue volume
Structural core	• Possess more of smaller amino acids • Less number of polar amino acids	• Possess less of smaller amino acids • Most the residues are polar in nature
Hydrogen bond	More of buried hydrogen bonds	Relatively less buried hydrogen bonds
Melting temperature (*T*_m_)	Higher	Lower
Hydrophobic cores	More tightly packed	Less compact
Ion pairs	Higher frequency	Lower frequency
Hydrogen bonding	Higher H‐bonding network	Moderate H‐bonding network
Surface loops	Shorter and rigid	Longer and flexible
Amino acid composition	Increased charged residues	Balanced composition
Δ*C*_p_	Lower	Higher
Δ*S*	Rigidity and hydrophobicity reduce the entropic gain	High gain upon unfolding
Δ*G*	Flat	Sharp
Cavity volume	Minimal	Higher
Arg/Lys ratio	High Arg	Balanced

Typically, proteins function in their native state, wherein the three‐dimensional configuration of polypeptide chains is designed to minimize the exposure of their hydrophobic core to the surrounding aqueous environment. Solvation, intramolecular interactions including van der Waals forces, hydrogen bonding and electrostatic nonbonding interactions, and local covalent bridges like disulfide bonds, etc. contribute to maintain the stability. The observed hydrophobic effect primary arises from the physiologically active structure of the protein. On the other hand, the entropic cost associated with the breaking of the hydrogen‐bond network is effectively counteracted by the presence of nonpolar residues. Such hydrophobic groups often form the core of the protein. Enthalpic interactions that bring changes at the atomic level also promote hydrophobic organization and aid to stabilize the folded state of proteins [20].

It is known that the reversible or irreversible transitions are influenced by various factors including temperature, chemical denaturants, pH, pressure, etc. Irreversibility typically results from aggregation or chemical modification and often follows first‐order reaction kinetics. The change in free energy Δ*G*_(FU)_, between the folded and unfolded states are governed by their thermodynamic stability. In case of two‐state model, a reversible transition between the states is observed without the formation of any intermediates. In general, Δ*G*_(FU)_ is determined by following the unfolding of proteins through chemically induced denaturation. The degree of unfolding at a given denaturant concentration [*D*] can be evaluated employing various methods like fluorescence and/or circular dichroism (CD). By analyzing the spectroscopic signals, the equilibrium constant (*K*) and hence the Δ*G*_(FU)_ can be evaluated as a function of [*D*].

For mesophilic proteins, Δ*G*_(FU)_ typically ranges from 30 to 60 kJ/mol, reflecting the energy of a few hydrogen bonds or hydrophobic interactions. Interestingly, at 25°C, the Δ*G*_(FU)_ for thermophilic proteins may not be higher than that of mesophilic proteins. The temperature at which the unfolded (U) and folded (F) states are equally populated (where *K* = 1 and Δ*G*_(FU)_ = 0) is known as the melting temperature (*T*_m_) and is an indicator of thermal stability of proteins. With Δ*G*_(FU)_ values across wide range of temperatures, it is possible to determine the enthalpy change (Δ*H*) and the difference in heat capacity (Δ*C*_p_) between the unfolded (U) and folded (F) states by analyzing the thermal unfolding processes [21] employing techniques like differential scanning calorimetry (DSC) and other spectroscopic methods, primarily CD and fluorescence. The insights obtained from these methods are often complementary. DSC provides the change in enthalpy of unfolding, denoted as Δ*H* ^cal^, which is independent of any specific model, while CD gives Δ*H* ^van’t^ ^Hoff^, which is specific for a two‐state model. These analyses collectively contribute to a comprehensive understanding of the thermodynamic properties associated with the unfolding of proteins as given in Equation ( (1)).

(1) $$d(\ln K)/d(1/T)=–\Delta H^{\mathrm{van}’\mathrm{tHoff}}/R$$

The ratio of Δ*H* ^cal^:Δ*H* ^van’t^ ^Hoff^ ∼1 suggests two‐state models. The increase in Δ*H* with temperature is attributed to the fact that the unfolded states (U) possess a higher heat capacity as compared to the folded state (F).

(2) $$d(\Delta H)/dT=\Delta C_{p}$$

To determine Δ*G*_(FU)_ as a function of temperature, it is essential to determine Δ*H* at specific temperatures, typically the melting temperature (*T*_m_), where Δ*H* is denoted as Δ*H*_m_. The difference in heat capacity (Δ*C*_p_) can be obtained from the slope of the linear plot of Δ*H*_m_ vs *T*_m_ (Equation (2)) [22].

With the values of *T*_m_, Δ*H*_m_, Δ*S*, and Δ*C*_p_, Δ*G*_(FU)_ as a function of temperature can be obtained using the modified Gibbs–Helmholtz Equation (3).

(3) $$\Delta G_{\left(\mathrm{FU}\right)}(T)=\Delta H_{m}–T\Delta S_{m}+\Delta C_{p}[(T‐T_{m})‐T\ln(T/T_{m})]$$

Hence, at maximum temperature, *T*_max_, where the thermodynamic stability is maximum, Δ*S* = 0. Thus, at *T*_max_, Δ*G*_(FU)_ is solely influenced by its enthalpic contributions. The curvature of the Δ*G*_(FU)_ (T) depends on Δ*C*_p_, as indicated in Equation (4) [23].

(4) $$d^{2}(\Delta G)/dT^{2}=–\Delta C_{p}/T$$

In simpler terms, a smaller Δ*C*_p_ results in a flatter curve and a higher *T*_m_. It is suggested that Δ*C*_p_ is approximately a linear function of the number of amino acids in the protein and is also related to the hydrated surface area upon unfolding (accessible surface area—ΔASA). The relationships between Δ*C*_p_, the number of amino acids, and ΔASA provide different insights into the same physical phenomenon, highlighting the effects of protein size and the characteristics of the hydrophobic core [20, 24].

## 4 Kinetic Stability

The irreversible thermal unfolding observed in most proteins is primarily induced through aggregation or chemical modification of unfolded states (U). Therefore, equilibrium thermodynamics alone may not sufficiently describe these processes. A standard model for irreversible unfolding can be given as

(5) $$F\overset{K_{u}}{\underset{K_{f}}{⇌}}U\overset{K_{i}}{\to }X$$

where *F* is the native state, *U* is the reversibly unfolded intermediate, and *X* is the irreversibly inactivated state. Here, *k*_u_ and *k*_f_ are the rate constants for unfolding and refolding, respectively, and *k*_i_ is the rate constant for the irreversible step.

Applying the steady‐state approximation to state *U*, the observed rate of irreversible unfolding (*k*_obs_) is defined as

$$k_{\mathrm{obs}}=\frac{k_{u}*k_{i}}{k_{f}+k_{i}}$$

The stability is often quantified by the half‐life, *t*_1/2_ = ln2/*k*_obs_. In the kinetic regime that is typical of high physiological temperatures, where *k*_i_ >> *k*_f_, *k*_obs_ approximates *k*_u_. Hence, the overall stability will be limited by the rate of unfolding rather than the thermodynamic equilibrium. Therefore, maintaining low *k*_u_ will be a vital adaptation strategy for thermophilic proteins to ensure stability in vivo. Importantly, *k*_u_ values for thermophilic proteins are often several orders of magnitude lower than those of their mesophilic counterparts, even when their thermodynamic stabilities (Δ*G*) at ambient temperatures are comparable [23, 25].

## 5 Comparison of Thermophilic and Mesophilic Proteins

The distinct difference between the thermophilic and mesophilic proteins is not just a matter of higher melting temperatures (*T*_m_), but a fundamental difference in the way they manage to interplay between stability and biological function [26].

The thermodynamic profiling of proteins can be performed through the derived relationship between Δ*G* and Δ*C*_p_. The most critical thermodynamic difference lies in the curvature of the stability curve. The mesophiles typically exhibit a sharp, parabolic Δ*G*(*T*) curve with a high Δ*C*_p_ value. This results in a narrow temperature window for stability and makes the protein susceptible to both heat and cold denaturation. In thermophiles, the evolutionary pressure has favored a reduction in Δ*C*_p_ value that flattens the Δ*G* curve. This flattening of curvature allows the protein to maintain a relatively constant Δ*G* across a broad thermal range. In a recent study, it was suggested that this flattening was achieved through reduced hydration of nonpolar groups during unfolding that further effectively tune the solvent–protein interface [23, 27, 28].

The following structural strategies can be adapted to compare the proteins’ core packing and surface networking. While both protein types share the same fold of families, their internal architectures differ significantly. The thermophilic proteins feature a fractal‐like packing of their core. By replacing larger residues with branched nonpolar amino acids (Ile, Val), they maximize van der Waals contacts and eliminate internal cavities. While in mesophiles, the ion pairs are often solvent‐exposed and offer only local stabilization [29, 30]. In thermophiles, these are organized into extended networks that can act as molecular binders to prevent the cooperative collapse of surface loops. Moreover, the thermophilic proteins consistently show a higher density of interactions between the residues that are far apart in the primary sequence but get closer in three‐dimensional space [31, 32]. A common misconception is that thermophilic proteins are better because they are more rigid. Critically, at their respective optimal growth temperatures, thermophilic and mesophilic proteins often exhibit identical root‐mean‐square fluctuations [33]. A thermophilic enzyme at higher temperature possesses the same degree of flexibility required for catalysis as its mesophilic homolog at lower temperature [34]. At room temperature, the thermophilic proteins are too rigid to function whereas at high temperatures, the mesophilic proteins are too flexible, leading to entropic unfolding [32]. Literature reports are available on the utilization of ML approaches to identify specific amino acid substitution patterns that define the transition from mesophiles to thermophiles [35]. For example, arginine is preferred in thermophiles, as the guanidinium group forms multiple hydrogen bonds and ion pair interactions, whereas lysine is more entropically costly at high temperatures [36, 37]. Similarly, in thermophiles, proline is frequently incorporated in loops to decrease the configurational entropy of the unfolded state, thereby increasing the kinetic barrier to unfolding (*k*_u_) [38, 39].

Thermophiles and mesophiles represent two different classes of proteins that exhibit different temperature optima for their functioning. The primary difference between them lies in their adaptation to specific temperature ranges. A comparison of various factors based on their inner core is summarized in Table 1.

## 6 Stabilizing Factors of Thermophilic Proteins

**FIGURE 2:**
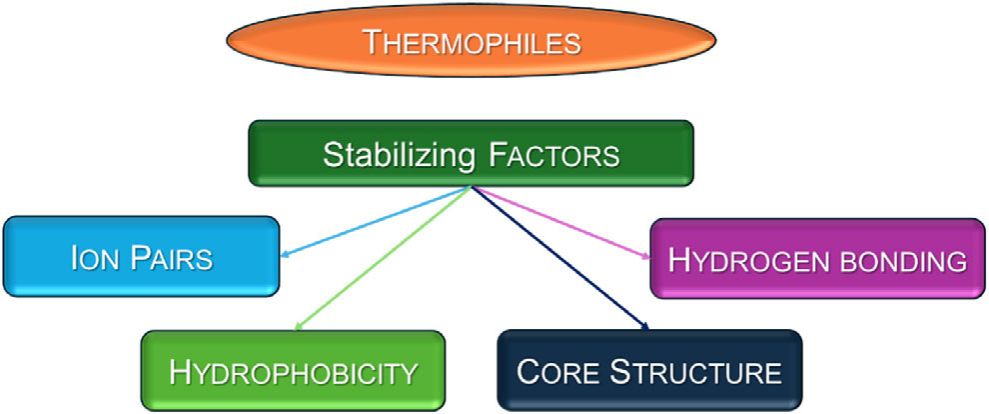
Stabilizing factors for thermophilic proteins.

There are several factors that have been identified and proven to stabilize the proteins, particularly, thermophiles at higher temperatures (Figure 2). Some of the stabilizing factors are briefed as follow.

## 7 Ion Pair Abundance

Thermophilic proteins that undergo complete reversible unfolding are often small and monomeric with a single structural domain, while the mesophilic proteins may vary in size and oligomeric structures. Recent investigations have revealed the key structural distinctions between thermostable proteins and their mesophilic counterparts, further to provide insights into the observed enhancement of thermal stability. One of the notable structural differences is the increased number of ion pairs in thermophilic proteins as compared to the mesophiles [32, 40]. The number of ion pairs is positively correlated with thermal stability. Extremely thermophilic proteins demonstrate stronger ion pair interactions often at distances of less than 4 Å from each other, while mesophilic proteins typically have separations ranging from 6 to 8 Å [41].

Research by Szilagyi and Zavodszky, as well as Gromiha et al., supported the idea that a high number of ion pairs contribute to the stability of thermophiles [42, 43]. There exist speculations contradicting the above statement that increased number of ion pairs in thermophilic proteins is compatible with higher average hydrophobicity [44, 45]. In thermophilic proteins, nonionic residues are surrounded by much higher levels of hydrophobic residues as compared to those in mesophilic proteins. Moreover, ion pairs donate to the increased rigidity of tertiary structures which in turn enhances the stability and induce tight packing, along with slower unfolding capability as compared to mesophilic counterparts [42, 45].

## 8 Hydrophobicity of Buried Amino Acid Side Chains

When comparing the amino acid composition of mesophilic and thermophilic proteins, it is evident that the former is considered by larger residue volumes, increased residue hydrophobicity, a greater abundance of charged amino acids (notably glutamic acid, arginine, and lysine), and a lower occurrence of uncharged polar residues (especially serine, threonine, asparagine, and glutamine) [46, 47]. Recent studies have highlighted the relationship between amino acid composition and thermostability, demonstrating that the nature of amino acid side chains significantly influences the thermal stability [48, 49]. Despite the similarities in the polar and nonpolar contributions to surface area and density between these proteins, the thermophiles have additional hydrophobic buried amino acid side chains [44]. The hydrophobicity around amino acid side chains, assessed within 8 Å radius, is higher in thermophilic proteins that further leads to tight residue packing during protein folding. This tight packing enhances resistance to unfolding and aggregation. In addition, thermophilic proteins have a greater number of regular hydrophobic internal residues than the mesophilic proteins [23].

## 9 Structural Cores

Meruelo et al. have reported thermophilic proteins to possess a significant percentage of smaller amino acids such as glycine, alanine, serine, and valine and fewer larger and/or polar amino acids like cystine, aspirin, glutamic acid, glutamine, and arginine [50]. Moreover, the tight packing during folding increases resistance to unfolding and aggregation due the presence of fewer large and reactive side chains. This finding is consistent with previous reports on the observed tight packing of interior residues in thermophilic proteins [51]. On the other hand, though thermophilic proteins have hydrophobic and longer side chains residues, they are strategically positioned within the interior of the tertiary structure to maximize residue–residue interactions, further to result in a tight, stable, and compacted structure [52]. It was also reported that hyperthermophilic proteins possess substantially greater number of disulfide bonds than mesophilic and thermophilic proteins. This emphasizes the necessity for a compact tertiary core to enhance the stability of proteins [50, 53, 54]. These characteristics can assist in the process of optimizing the close packing of core residues that can further enhance the stability of the tertiary structure. All these supports the fact that precise arrangement of residues can enhance the communications between adjacent residues which can further boost thermal stability.

## 10 Hydrogen Bond Distribution

Thermophilic proteins display distinct hydrogen bond distributions as compared to mesophilic proteins [32]. In thermophilic proteins, 49% of the hydrogen bonds connect the buried core residues with those of the exposed residues, whereas only 42% exhibit this pattern in mesophilic proteins [42]. The observed hydrogen bonds in thermophiles are between the main and the side chain residues. This distinguished hydrogen bond distribution in thermophilic proteins contribute to enhanced stability by anchoring the vulnerable exterior to the compact core, thereby crosslinking all the elements of the tertiary structure [55]. These structural differences collectively contribute to the increased rigidity and stability observed in thermophilic and hyper‐thermophilic proteins, making them well‐suited for functioning at high‐temperature environments [56].

## 11 Molecular Adaptation Strategies

Thermophilic proteins are known to achieve stability synergistically through structural features that increase conformational rigidity and resist thermal unfolding. Unlike mesophilic proteins, which rely more on surface exposed hydrogen bonding and electrostatic interactions that suit for moderate temperatures, thermophiles prioritize internal stability through a more robustly packed hydrophobic core and extensive long‐range interaction networks. The following are key integrative features of molecular adaptation [57, 58].

**FIGURE 3:**
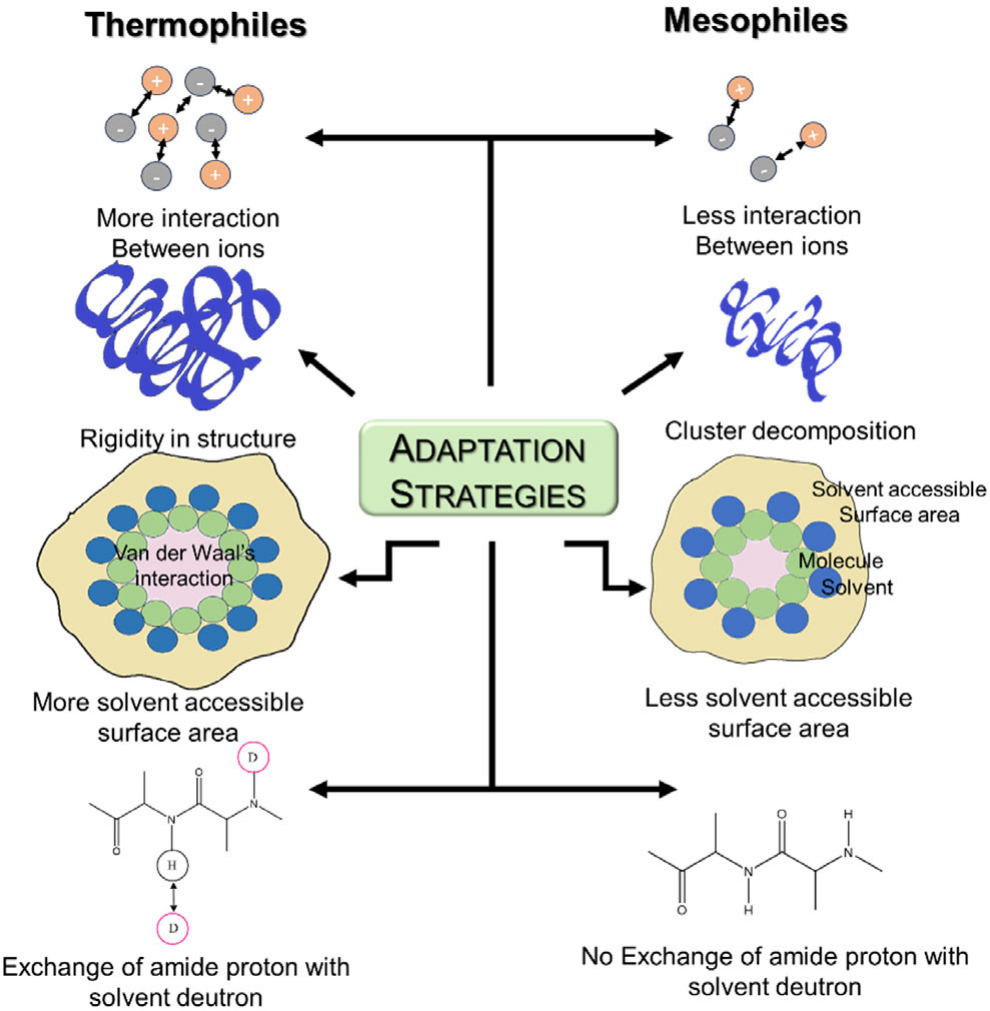
Comparison of strategies and structural determinants of proteins to adapt themselves to high temperature conditions and exhibit their functions.

Thermophilic proteins employ multiple strategies to exhibit their functions under high thermal stress. These adaptations do not occur independently but represent an overall optimization of the protein's energy landscape [59]. The various strategies (Figure 3) that are adapted by thermophiles are discussed as follows.

*Thermodynamic Strategies*—The primary thermodynamic objective for thermophilic proteins is to maintain a positive Gibbs free energy of folding (Δ*G*_fold_) at elevated temperatures. Thermophiles often exhibit low heat capacity change upon unfolding (Δ*C*_p_) as compared to mesophiles. This results in a flat Δ*G*(*T*) curve, extending the temperature range where the protein remains folded [22]. In the case of thermophilic RNase H, the achieved *T*_m_ is higher than its *Escherichia coli* homolog by 20°C [60]. This could primarily be explained through the above mechanism. On the other hand, the entropy increases as water molecules are released during folding and further compensates for the loss of conformational entropy at high temperatures.

*Kinetic Strategies*—Kinetic stability is defined by the height of the energy barrier to unfolding (*k*_u_). In many thermophilic systems, stability is achieved not by making the folded state more stable than the unfolded state (Δ*G*), but by making the *transition* to the unfolded state extremely slow (*k*_obs_ ≈ *k*_u_). In the case of ferredoxin that is more prone to chemical decomposition, the rightward shift is observed in the stability curve which further ensures that the protein survives long enough to perform its biological role [23].

*Structural Strategies*—Structural adaptations focus on maximizing internal connectivity and minimizing loose elements. Unlike the isolated ion pairs in mesophiles, thermophilic proteins utilize extensive, short‐range (<4 Å) ion‐pair networks that cross‐link the protein surface to its core. Thermophiles often prioritize nonpolar, smaller amino acids (Gly, Ala, and Val) to eliminate internal cavities, to result in a more compact and rigid hydrophobic core [61, 62].

*Dynamic Strategies*—The dynamic behaviors of thermophilic proteins are characterized by the display of same flexibility at higher temperatures that mesophiles show at lower temperatures. Moreover, the free energy landscape of thermophilic proteins resembles a narrow and deep funnel that limits conformational diversity and limits the side‐chain fluctuations to prevent blocking of active sites at higher temperatures [63, 64].

*Evolutionary Strategies*—Evolution has optimized protein sequences to remove weak links. There is a notable evolutionary trend toward replacing thermolabile residues like methionine, which is susceptible to oxidation, with more robust hydrophobic or charged residues. Evolutionary pressure has favored increased long‐range interaction densities, allowing for global stability to emerge from local structural enhancements [65].

Thermal resilience is known to positively correlate with the frequency and strength of ion pairs. In extreme thermophiles, these interactions often occur at shorter distances (<4 Å) compared to mesophiles (6–8 Å), contributing to tertiary structure rigidity and slower unfolding kinetics. The stability can further be enhanced by the abundant presence of smaller, nonpolar amino acids (e.g., Gly, Ala, and Val) that can facilitate tighter packing and minimize internal cavities. This compact architecture is cross‐linked by a distinct hydrogen bond distribution, where a higher percentage of bonds (49% in thermophiles vs. 42% in mesophiles) connect buried core residues to the exterior. Moreover, the adaptation involves the presence of an enriched aromatic and nonpolar residues that strengthen the hydrophobic driving force for folding, while simultaneously reducing thermolabile residues like methionine, which are susceptible to oxidation at high temperatures [56, 61, 62].

Figure 3 shows the structural determinants that contribute to thermostability, including enhanced hydrophobic packing, increased ion‐pair networks, and improved hydrogen bonding interactions. These features collectively reduce conformational flexibility and stabilize the folded states at elevated temperatures.

A comparative study of RNAse from closely related mesophilic and thermophilic enzymes (*Thermus thermophilus* and *Escherichia coli*) has revealed interesting differences [66]. Despite having similar sizes and expected variations in the available surface area upon unfolding, the former variant revealed a lower Δ*C*_p_ (7.5 kJ/mol/K) as compared to the later (11.3 kJ/mol/K). This results in a flat Δ*G*_(FU)_ (*T*) curve, contributing to a 20°C higher *T*_m_ for the thermophilic enzyme (86°C vs. 66°C). Notably, in the functional temperatures of their respective organisms, both enzymes have similar Δ*G*_(FU)_ values. Rubredoxin from *Pyrococcus furiosus* (pRdx), a well‐studied monomeric protein, have also displayed irreversible thermal unfolding [66]. Its thermodynamic stability, however, can be assessed through the continuous formation and breaking of hydrogen bonds, at different temperatures. These findings provide a better understanding on the stability and flexibility of proteins and also provide insights into the underlying biological processes of their activity [67].

**FIGURE 4:**
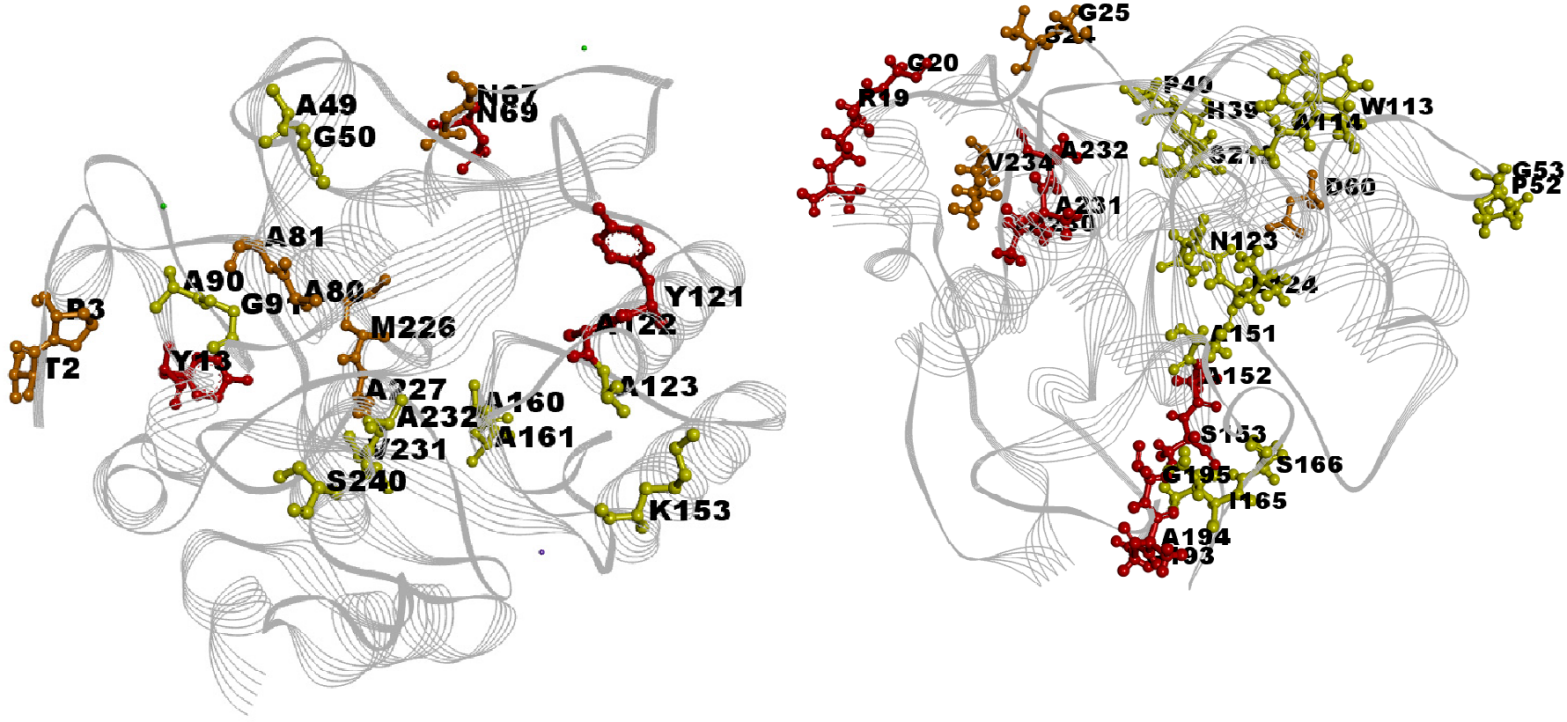
Three‐dimensional structures of mesophilic 1GCI and thermophilic 1THM bacterial protease.

Previously investigations employing vibrational energy diffusivity method have revealed that the vibrational energy transfer within proteins plays key roles in diverse biological processes [68]. Persistent fluctuations in both thermophilic and mesophilic proteins were observed through molecular dynamic simulations when conducted at different temperatures—308, 325, and 350 K. To further deepen the understanding on system stability, frequency‐resolved communication maps were generated for mesophilic 1GCI and thermophilic 1THM bacterial protease, to identify the key residues that were involved in energy transfer pathways. Highly active residues that play a significant role in either stabilizing or destabilizing proteins are thus identified and represented in Figure 4. This compositional difference indicates that thermophilic proteins have more extensively on nonpolar (hydrophobic) residues, which contribute to a stronger hydrophobic core packing and enhanced internal stability at elevated temperatures. Conversely, the increased presence of polar and charged residues in the mesophilic protein suggested a greater dependence on surface hydrogen bonding and electrostatic interactions, which are effective at moderate temperatures but less stable under high‐thermal stress. Thus, the correlation between residue composition and protein stability supports the observation that thermophilic proteins enhance thermal resilience through hydrophobic stabilization, whereas mesophilic proteins maintain structural integrity primarily through polar and electrostatic interactions. Additionally, the influence of salt bridges and noncovalent interactions on protein stability were also investigated to reveal valuable insights into the intricate relationship between protein activity and thermal resistance, further to provide a comprehensive understanding of how thermophilic and mesophilic proteins behave at different temperatures. These findings have revealed the underlying mechanisms of protein flexibility and stability, that further govern their biological functions [68].

Molecular dynamic simulations were carried out to understand the thermal stability of Glycosyl hydrolases that are stable and active across a wide range of temperatures. Investigations have revealed that not all salt bridges contribute to thermal stability [69]. The movement of amino acid side chains was revealed to be a key factor. The increase in the mobility of side chains at high temperatures blocks the active site and deactivates the enzymes. Therefore, it was shown that the flexibility and not just rigid structures like salt bridges dictates the functioning of enzymes at higher temperatures [70].

**FIGURE 5:**
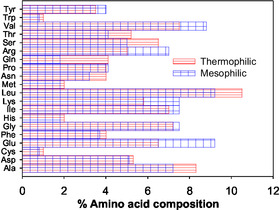
The percentage composition of amino acids in thermophiles and mesophiles from diverse families.

Thermophilic proteins have higher levels of charged and hydrophobic residues. A neural network effectively differentiates the thermophiles and mesophiles based on side‐chain mobility. At high temperatures, mesophilic enzymes are more flexible and their functions are blocked, while thermophiles remain stable. The ratio of calculated distance‐dependent statistical potentials was used to computationally distinguish the two protein types [71]. Figure 5 shows the percentage composition of amino acids in thermophiles and mesophiles from diverse families.

Recently several structural factors that contribute to heat stability of thermophiles were revealed [31,72, 73]. Proteins with greater shape and more branch point locations in their amino acid side chains showed enhanced thermostability. The Gibbs free energy of hydration for the native proteins showed an inverse relationship with thermostability. The study concluded that better packing for structural stability and optimal solubility for proper functioning of proteins is required in a balanced manner to achieve maximum overall thermal stability [74].

## 12 Optimizing Amino Acid Compositions and Coupling Patterns in Thermophilic Proteins

**FIGURE 6:**
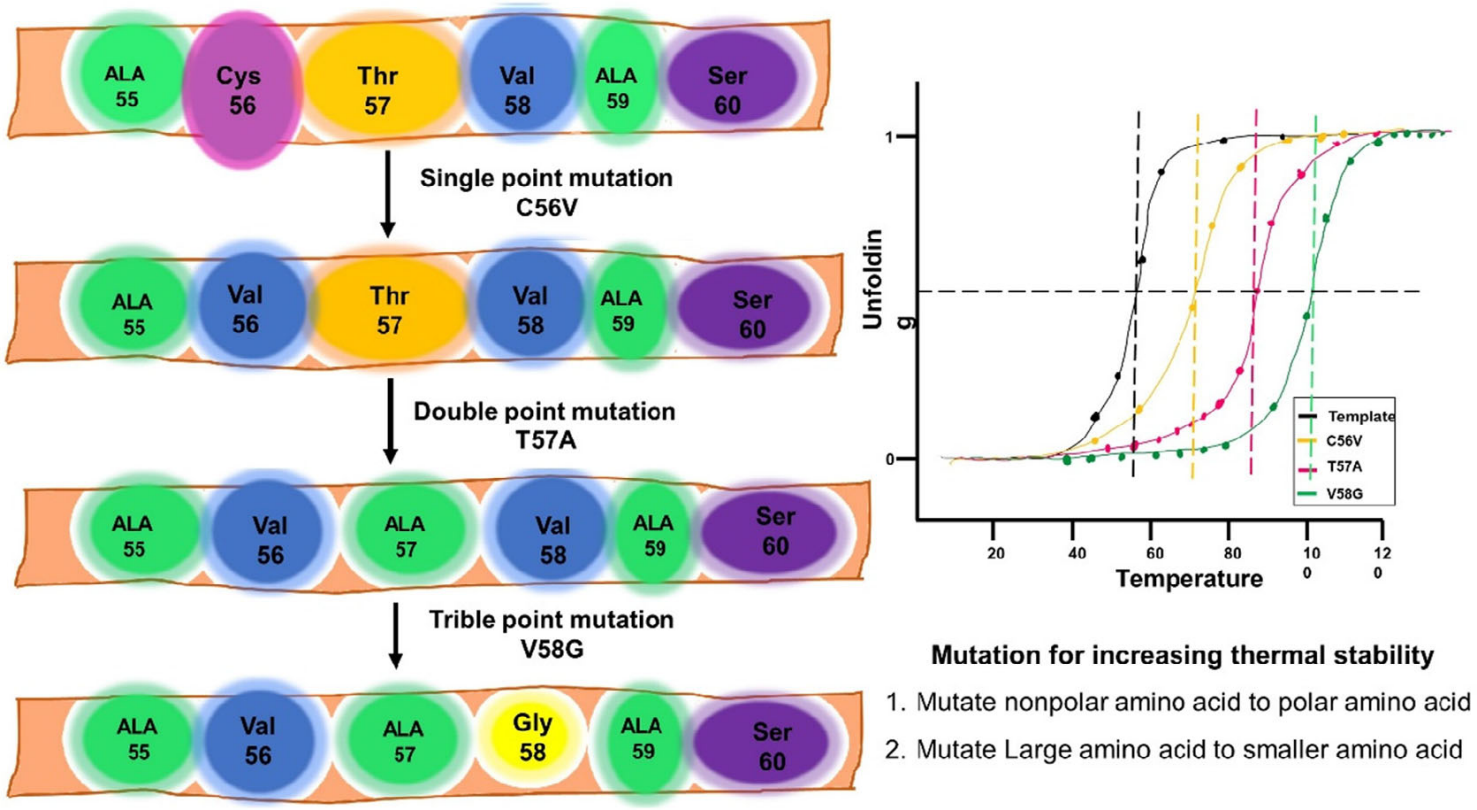
The role of specific amino acid residues in the stability of thermophilic proteins.

Extensive research was conducted to understand the role of certain amino acids in the stability of thermophilic proteins (Figure 6). Site‐directed mutagenesis was performed and evaluated for their stability variations. By analyzing the composition of amino acids, it is possible to streamline the significance of each kind of amino acids. However, this approach does not account for the interactions or coupling effects that essentially enhances thermal stability. Further, to explore these coupling patterns, structural analyses were performed to identify the features that enhance thermal stability. A novel statistical approach revealed the sequence‐based investigations on the composition of amino acids to offer valuable yet basic insights into amino acids related to thermal stability [75].

A technique was developed to identify significant amino acid coupling patterns in thermophilic proteins, focusing on pairs separated by intervening residues and revealed the frequency bias between thermophiles and mesophiles using the ϕ value [76]. The results showed a notable bias in these patterns, primarily driven by temperature adaptation, which can be used to distinguish thermophilic proteins, especially when structural data are limited. Furthermore, the prevalence of these statistically significant coupling patterns has shown a strong association with optimal growth temperatures [76].

Investigation on the enhancement of heat resistance and thermal stability in H167L mutant of yeast MgeI revealed the enhancement of Hsp70‐mediated protein refolding. The flexibility of the mutant protein was revealed through heat denaturation, ionic quenching, and restricted proteolysis. Moreover, the investigations have revealed a comparatively more rigid structure. The stiffness and hydrophobicity of the above mutant was found to be structurally and functionally similar to thermophilic proteins [77].

Another interesting finding revealed that the application of recombinant aspartic protease BsAPA from *Pichia* *pastoris* was limited by heat inactivation. Efforts to enhance the thermal stability were attempted by mutating its autocatalytic site, as this site in the aspartic protease is known to diminish its thermal stability. Therefore, site‐directed mutagenesis near the autocatalytic site was attempted as an efficient strategy to enhance stability [78]. Similar mutational studies were carried out to investigate the biochemical properties of recombinant glucose isomerase. Enhanced activity was observed with the mutants in the presence of certain metal ions like Co^2+^, Cu^2+^, and Mn^2+^ than the recombinant protein [79].

Similarly, the effect of specific amino acid substitutions on the temperature stability of xylanase was also investigated. Herein, the mutations caused structural changes in the enzyme, rendering stiffness to the side chains and the fill the grooves, ultimately to leading to enhanced stabilization and improved thermostability of the mutants [80].

Dynamic processes crucial for the biological functions of mesophilic and thermophilic ribonuclease H (RNase H) enzymes were recently examined [81]. Both enzymes displayed similar kinetic rate constants for transitions between their major and alternate conformational states. However, the stability difference was less pronounced in thermophiles as compared to mesophilic RNase H. By mutating a conserved glycine residue, the mesophilic RNase H was able to replicate the thermodynamic equilibrium seen in thermophiles that further helps to introduce more intrahelical hydrogen bonds in the thermophilic RNases H [81].

## 13 Engineering Strategies for Enhancing Thermostability

Rather than relying on exhaustive site‐directed mutagenesis, modern protein engineering approaches employ targeted strategies to reinforce the structurally weak links of mesophilic templates. These include the core packing and the cavity filling strategies. As compared to natural adaptations, these engineering efforts mainly focus on identifying internal voids in mesophilic proteins and fill them with bulkier hydrophobic residues (e.g., Leu and Phe). For example, in the engineering of T4 lysozyme, the introduction of hydrophobic residues into its internal cavities resulted in an elevation of *T*_m_ by 10°C that has resulted from enhanced van der Waals contacts that further drives its stability. In the case of the structure‐based approach B‐Factor‐guided rigidification (B‐Fit), the residues with high B‐factors are targeted [82, 83]. By mutating these flexible residues with more conformational entropy‐restricted residues or by introducing local ion pairs, these proteins are rigidified against thermal fluctuations. For example, the stabilization of *Bacillus subtilis* lipase A was achieved by targeting the most flexible loops identified via B‐factor analysis, significantly increasing its half‐life at 60°C without compromising its catalytic turnover [84].

The sequence‐based consensus design and the conventional reconstruction strategy assume that the residues conserved across thermophilic homologs contribute to stability. By aligning sequences and selecting the most frequent consensus amino acids, it is possible to create highly stable chimeric proteins [85, 86]. For example, designing the consensus phytase enzymes with melting temperatures exceeding 90°C was produced to outperform any naturally occurring variant. This demonstrates the power of evolutionary signatures in defining thermal resilience [87]. For entropic stabilization through loop shortening and proline introduction, it was suggested that introducing proline into loops or the second position of α‐helices decreases the entropy of the unfolded state (Δ*S*_unfold_), thereby increasing Δ*G*_fold_ [88]. Similarly, shortening long disordered loops reduces the entropic penalty of folding. In the engineering of α‐amylase, the combination of loop shortening and proline substitutions has resulted in a synergistic increase in thermostability, allowing the enzyme to function in high‐temperature environments of industrial starch processing [89]. Finally, with the modern computationally directed evolution frameworks like FRESCO (Framework for Rapid Enzyme Stabilization by Computational libraries), it is possible to screen thousands of virtual mutations using molecular dynamics and Rosetta‐based energy calculations before experimental validation [90]. All these reduce the experimental workload and effectively bridge the gap between in silico predictions and industrial applications.

Thermophilic proteins are often considered as suitable scaffolds for numerous protein engineering applications as the native structure of these proteins displays higher intrinsic stability as compared to their mesophilic counterparts. This enhanced stability is generally attributed to structural features such as compact hydrophobic cores, stronger electrostatic interaction networks, and reduced conformational flexibility. Owing to these characteristics, thermophilic proteins are able to withstand a greater number of mutations without significant loss of structural integrity. Such mutational tolerance enables the introduction of catalytic or functional modifications while preserving the overall stability of the protein. Consequently, thermophilic proteins serve as promising starting templates for engineering enzymes with improved performance for industrial and biotechnological applications [55, 91, 92].

## 14 ML‐Based Approaches to Investigate Thermophilic Proteins

### 14.1 Comparison of Sequence vs. Structure‐Based Models

The sequence‐based model tools primary utilize amino acid sequences and evolutionary information like the position‐specific scoring matrices. They are highly scalable and suitable for screening genome‐wide stability where structural data are not available. However, they are often limited toward capturing the nonlocal coupling effects and three‐dimensional interactions like the ion‐pair networks that are critical for thermal resilience. On the other hand, the structure‐based models leverage three‐dimensional coordinates while evaluating features that include core packing density and buried hydrogen bond distributions. Though more accurate for predicting the effects of site‐directed mutations, their utility is limited by the availability of high‐resolution structures in databases like Protein Data Bank (PDB) [33].

### 14.2 Evolution of Algorithms: Classical ML to Deep Learning

The earlier tools of classical ML focused on hand‐engineered features such as amino acid composition and inter‐residue distance statistical potentials. Though these are easily interpretable, they are limited for their ability to capture complex, nonlinear dynamics of side‐chain mobility (examples: SVM, random forest, etc.). In the case of deep learning, modern architectures, including graph neural networks (GNNs) and transformer‐based models like ThermoMPNN, represent the current frontiers. Unlike traditional models, ThermoMPNN utilizes a message‐passing framework to learn the local environment of every residue, making it exceptionally capable to predict the effect of specific substitutions on Δ*G* and *T*_m_. These models effectively bridge the gap between structural rigidity and functional flexibility [93] (example: ThermoMPNN). A significant conceptual hurdle is the distinction between prediction targets. Moreover, the optimal growth temperature is an organismal‐level metric that correlates with average protein stability across a proteome, while the melting temperature (*T*_m_) is a protein‐specific thermodynamic threshold. Models trained on the former data often exhibit dataset bias and perform poor when applied to individual protein engineering tasks where *T*_m_ is the primary concern. Also, most training sets are heavily weighted toward mesophilic proteins, leading to diminished accuracy when predicting the extreme stability of hyperthermophilic scaffolds. Some of the modern tools and approaches employed in ML and DL, along with their advantages and applications are listed in Table 2.

**TABLE 2:** Summary of different models, their advantages, and applications.

Model/approach	Advantages	Uses
AutoProp	Automatically extracts useful sequence features and reduces the need for manual feature design. Works well with large protein datasets	Used for predicting whether a protein is thermophilic based on sequence information [94]
ProLaTherm	Uses optimized sequence descriptors and classification algorithms to improve prediction accuracy. Computationally efficient	Applied for identifying thermophilic and mesophilic proteins from amino acid sequences [95]
DeepTM	Deep learning framework capable of detecting complex patterns related to protein thermostability. Provides high prediction performance	Used for predicting protein melting temperature and thermal stability [96]
SAPPHIRE	Combines physicochemical and evolutionary features to improve reliability of predictions	Useful for screening proteins with potential thermostable properties [97, 98]
SCMTPP	Simple scoring‐based approach with good interpretability and low computational cost	Used for rapid classification of thermophilic proteins using dipeptide composition [99]
Convolutional neural networks (CNNs)	Able to identify local sequence patterns and motifs associated with protein stability. Requires minimal manual feature extraction	Applied to analyze protein sequences and detect stability‐related patterns [100]
Deep neural network (DNN) model	Learns complex nonlinear relationships among sequence features, improving prediction accuracy	Used in advanced predictive models for thermostability and protein classification [101]
ANOVA‐based feature selection	Selects important features and removes redundant data, improving model efficiency	Helps improve performance of machine learning models used for thermophilic protein prediction [102]
PseAAC (pseudo amino acid composition)	Represents both amino acid composition and sequence order information, giving richer sequence description	Widely used as an input feature for machine learning models in protein classification studies [76]
PROTS model	Integrates structural and sequence‐based information for improved prediction	Used for predicting protein stability and identifying thermophilic characteristics [103]
ELM (extreme learning machine)	Very fast training speed and simple network structure with good generalization ability	Applied in rapid prediction of protein thermostability using biological datasets [104]
TmPred	Employing a combined protein language model, graph convolutional network, and graphormer module, this model can predict the *T*_m_ of thermophilic protein	This deep learning model offers effecting mining and engineering thermophilic proteins by probing their *T*_m_ [105]
ProCeSa	Analyze how modifications like circular permutation affect protein folding and stability. Enables comparison of native and modified proteins to assess stability differences	To design proteins with modified topology, such as circularly permuted proteins. Helps tp understand proteins fold when their sequence connectivity is altered [106]
ESMStabP	Works directly from amino acid sequences, so no 3D structure is required. Provides reliable predictions of whether a mutation stabilizes or destabilizes a protein	Helps identify mutations that improve protein stability for industrial or research applications. To study the effect of amino acid substitutions, affect folding and stability [107]
JanusDDG	High accuracy even without 3D structure. Suitable for large‐scale mutation screening	Protein stability prediction, disease mutation analysis, rational protein design [108]
StableESM	Better prediction of stabilizing mutations, fixes scaling issues of large protein models	Mutation effect prediction, stability‐guided protein engineering [109]

### 14.3 Stability Prediction via ML: Addressing Dataset Limitations and Research Reproducibility

ML methods are now being widely utilized in the prediction of protein's thermal and mutational stabilities. Many of these models rely majorly on experimentally derived datasets that contain thermodynamic parameters such as melting temperature (*T*_m_) or changes in Gibbs free energy (Δ*G*). Databases such as ProTherm and FireProtDB have played an important role in enabling the development of predictive algorithms by providing curated collections of experimentally derived stability data [110].

Despite their significance, these datasets present several challenges that can influence the reproducibility and generalization of ML models. One major limitation is the availability of relatively smaller size of experimental datasets as compared to those used in other areas of ML. In addition, stability measurements are often obtained under different experimental conditions, including variations in pH, temperature, buffer composition, and measurement techniques. Such variability can introduce inconsistencies in the training data and may affect the reliability of model predictions [111, 112].

Another challenge arises from the uneven representation of protein families in stability datasets. Certain model proteins and enzyme classes were studied more extensively than others, which may also introduce bias ML models toward specific sequence or structural features. As a result, models trained on these datasets may perform well for proteins similar to those present in the training data but may struggle to generalize for unrelated protein families.

Reproducibility can also be influenced by differences in dataset preprocessing, feature engineering, and model evaluation strategies. For example, variations in the method of dataset filtering, handling redundant sequences, or development of training and test sets can lead to differences in reported performances across studies. To address these issues, the use of standardized benchmark datasets and transparent reporting of validation procedures is being encouraged. Such practices will help improve reproducibility and allow more reliable comparison of machine‐learning models developed for protein stability predictions [113, 114].

Recent computational modeling of small protein mutants like the Trp‐cage provides a molecular‐level view of the achievement of thermophilic stability through the modification of the folding–unfolding energy landscape. These simulations reveal that thermophilic adaptations often result in reduced changes in heat capacity (Δ*C*_p_) that further minimizes the temperature sensitivity of the Gibbs free energy. This curvature flattening of the Δ*G* profile is a primary strategy for maintaining structural integrity across a wide range of temperatures [115, 116].

In silico investigations into the thermodynamics of folding highlight the critical role of the solvent environment. Simulations suggest that thermophilic proteins optimize the excluded volume effect. By increasing the density of the hydrophobic core, these proteins minimize the entropy penalty associated with water restructuring at high temperatures. This suggests that the hydrophobic effect is not a static property but is dynamically modified through evolutionary amino acid substitutions to remain the dominant driving force for folding at extreme temperatures [117, 118, 119].

In another investigation, the heat capacity (*C*_p_) was employed to differentiate nonpolar and polar solvation effects and their impact on entropy and enthalpy. Protein unfolding generally results in a positive *C*_p_, which contributes to stability. Heat capacity has two primary contributions: hydration effects and protein–protein interactions. Theoretical analyses primarily address the hydration component, which explains most *C*_p_‐related characteristics that include the convergence of hydration entropy, the reduction in nonpolar hydration *C*_p_ at elevated temperature, and the temperature at which protein stability peaks [120].

Investigations on the protein dynamics and structure adjustments at elevated temperatures using molecular dynamic simulations have revealed two thermostable versions of the wild‐type enzyme and a para‐nitrobenzyl esterase [121]. The thermostable variants were found to stay closer to their crystal structures but exhibited greater fluctuations around their average structures. Additionally, they showed a slight but prominent increase in the radius of gyration. Analysis of vibrational density of states revealed that these thermostable mutants exhibit enhanced coordinated motions compared to the wild‐type. This adaptation to high temperatures appeared to limit large deviations from the native state while enhancing smaller scale fluctuations, which contributed to both entropy and stability. The most significant changes in dynamics were observed in surface loops, whereas other regions remained stable [121].

## 15 Conclusions

In conclusion, thermophilic proteins represent a remarkable model system for understanding molecular adaptation to extreme environments and for harnessing their potential in various fields of applied sciences. Their unique structural features ranging from optimized hydrophobic cores, enriched ion‐pair interactions, and altered hydrogen bond distributions to adaptive amino acid compositions collectively support their extraordinary thermal stability. Comparative analyses with mesophilic counterparts highlight the delicate balance between structural rigidity and functional flexibility that enables thermophiles to thrive at elevated temperatures. Advances in site‐directed mutagenesis, computational simulations, and in silico investigations have extended our mechanistic understanding, though complex amino acid coupling effects and long‐range interactions that remain an area for further investigation.

Beyond fundamental insights, thermophilic proteins serve as robust scaffolds for protein engineering, offering mutational resilience and versatility for industrial, pharmaceutical, and nanotechnological applications. The integration of ML with experimental data has emerged as a powerful frontier, enabling the prediction of thermal stability, functional classification, and design of novel variants with unprecedented accuracy. Together, these multidisciplinary approaches have expanded the scope of rational protein design and biocatalyst development.

Future investigations must continue to bridge the gap between existing experimental and computational methodologies, with a focus to unravel the interplay of amino acid networks, refine predictive algorithms, and engineer thermophilic proteins with tailored properties. This will not only enhance the knowledge of protein evolution and stability but also unlock innovative applications across biotechnology, medicine, and sustainable industrial processes.

Although substantial progress has been made in understanding thermophilic protein stability, several aspects remain feebly investigated. In particular, the balance between thermodynamic stability and kinetic resistance toward unfolding continues to be an area of active investigation. At the same time, computational approaches, including ML methods, have improved the ability to predict stability trends, but still, challenges related to limited training datasets and model interpretability exist. Addressing these issues will require stronger integration between experimental studies, structural analysis, and data‐driven computational approaches.

Overall, the comprehensive study of thermophilic proteins and their adaptation to extreme temperatures and the thermodynamics of their proteins contribute not only to fundamental scientific knowledge but also have practical implications that include the development of industrial processes and biotechnological applications.

## Conflicts of Interest

The authors declare no conflicts of interest.

## Data Availability

The data that support the findings of this study are available on request from the corresponding author. The data are not publicly available due to privacy or ethical restrictions.
